# Vocal tract anatomy of king penguins: morphological traits of two-voiced sound production

**DOI:** 10.1186/s12983-020-0351-8

**Published:** 2020-01-30

**Authors:** Hannah Joy Kriesell, Céline Le Bohec, Alexander F. Cerwenka, Moritz Hertel, Jean-Patrice Robin, Bernhard Ruthensteiner, Manfred Gahr, Thierry Aubin, Daniel Normen Düring

**Affiliations:** 1 Centre Scientifique de Monaco, Département de Biologie Polaire, 98000 Monte Carlo, MC Monaco; 2 Université de Strasbourg, CNRS, IPHC UMR 7178, F-67000 Strasbourg, France; 3 Institut des NeuroSciences Paris-Saclay (Neuro-PSI), UMR 9197 (CNRS, Université Paris XI), Orsay, France; 4 Department of Electronic Systems, Norwegian University of Science and Technology, 7491 Trondheim, Norway; 5 SNSB-ZSM Bavarian State Collection of Zoology, Section Evertebrata varia, Münchhausenstraße 21, 81247 Munich, Germany; 6 Department of Behavioral Neurobiology, Max Planck Institute for Ornithology, Seewiesen, Germany; 7 Institute of Neuroinformatics, ETH Zurich & University of Zurich, Zurich, Switzerland; 8 Neuroscience Center Zurich (ZNZ), Winterthurerstrasse 190, 8057 Zurich, Switzerland

**Keywords:** Syrinx anatomy, Micro computed tomography, 3D reconstruction, Spheniscidae, Vocal communication

## Abstract

**Background:**

The astonishing variety of sounds that birds can produce has been the subject of many studies aiming to identify the underlying anatomical and physical mechanisms of sound production. An interesting feature of some bird vocalisations is the simultaneous production of two different frequencies. While most work has been focusing on songbirds, much less is known about dual-sound production in non-passerines, although their sound production organ, the syrinx, would technically allow many of them to produce “two voices”. Here, we focus on the king penguin, a colonial seabird whose calls consist of two fundamental frequency bands and their respective harmonics. The calls are produced during courtship and for partner and offspring reunions and encode the birds’ identity. We dissected, μCT-scanned and analysed the vocal tracts of six adult king penguins from Possession Island, Crozet Archipelago.

**Results:**

King penguins possess a bronchial type syrinx that, similarly to the songbird’s tracheobronchial syrinx, has two sets of vibratory tissues, and thus two separate sound sources. Left and right medial labium differ consistently in diameter between 0.5 and 3.2%, with no laterality between left and right side. The trachea has a conical shape, increasing in diameter from caudal to cranial by 16%. About 80% of the king penguins’ trachea is medially divided by a septum consisting of soft elastic tissue (septum trachealis medialis).

**Conclusions:**

The king penguins’ vocal tract appears to be mainly adapted to the life in a noisy colony of a species that relies on individual vocal recognition. The extent between the two voices encoding for individuality seems morphologically dictated by the length difference between left and right medial labium. The septum trachealis medialis might support this extent and could therefore be an important anatomical feature that aids in the individual recognition process.

## Background

Birds exhibit a huge variety of vocal sounds and have exploited the use of acoustic signals in many behavioural contexts, e.g. mate choice and territory defence (reviewed in [1–3]). The characteristics of the sounds produced, the physical mechanisms of sound production and the underlying anatomy of avian vocalisations have been studied in many bird species [1–9]. While mammals produce sound in the larynx, birds have evolved a novel organ for sound production called the syrinx [10–12]. Despite different evolutionary origins of larynx and syrinx, the underlying physical mechanism of sound production in both mammals [13, 14] and birds [9] is based on self-sustained oscillations of the vibratory tissues caused by pressure differences of expiratory air-flow [13]. This myoelastic-aerodynamic (MEAD) principle allows biological systems to produce acoustic signals solely based on air flow and does not require direct neurological input or muscular control [9, 14]. However, in addition to MEAD-based sound production, many of the often complex sounds produced by birds and their specific acoustic characteristics require precise input of highly specialized muscles that affect the timing and spatial characteristics of the oscillations [15, 16]. In addition to musculature, cartilaginous structures and ossified syringeal elements can play an important role in the biomechanics of sound production, e.g. by impacting the position of the vibrating tissues [4, 17, 18].

Similar to the tissues present in the vocal apparatus of terrestrial mammals, the syrinx and trachea of birds mainly consist of four anatomical elements: bone, cartilage, muscle and vibratory soft tissue. The vibrating tissues of birds are analogous to the vocal folds of the larynx in mammals [19] and typically consist of medial labia (ML), medial tympaniform membranes (MTM) and lateral labia (LL) [20–22]. Goller and Larsen [22] experimentally disabled the MTM in zebra finches *Taeniopygia guttata* and northern cardinals *Cardinalis cardinalis* and concluded that ML and LL are most likely responsible for sound production. In addition to the often highly specialized anatomy of the syrinx, the respiratory tract [23], oropharyngeal-esophageal cavity [24], larynx, tongue [25], and beak also influence the sound production [26].

The detailed anatomy, i.e. the number and location of membranes, morphology of ossified elements as well as the number of muscles and their exact insertion sites on the syrinx differs immensely between species and adds to the intricacy of produced sounds (reviewed in [2, 6, 23, 27]). The general location of the vibratory soft tissues defines the syrinx into one of three types, tracheal, bronchial or tracheobronchial. While bronchial and tracheobronchial syrinx types have one set of ML and LL in each bronchus, tracheal type syrinxes have only one set of ML and LL in the trachea. Both the tracheobronchial and the bronchial syringes allow birds to produce two sounds simultaneously as vibrations can potentially be created independently at each bronchus [28]. Songbirds are able to produce sounds of different frequencies independently in each bronchus [29, 30] owing to their complex intrinsic syringeal musculature [4, 31] and lateralization in size of the vibratory tissues as reported for European starlings *Sturnus vulgaris* [32], white-crowned sparrows *Zonotrichia leucophrys* and zebra finches [33]. Only few studies also demonstrated the ability to simultaneously produce two sounds in non-songbirds, e.g. wood ducks *Aix sponsa* [34] and the Greater sage-grouse *Centrocercus urophasianus* [35], that produces a “double whistle”. Investigating the vocal tract in non-vocal learners (non-passerines) may allow us to identify direct links between specific acoustic features such as dual-sound production and the underlying morphological mechanisms, because we can assume that the vocal output is more directly linked to anatomical constraints [36–38].

The display calls of king penguins *Aptenodytes patagonicus* have been shown to be emitted during mate choice [39] and sex-differences in the fundamental frequency of the calls and the syllable pattern have been identified [40]. Furthermore, playback experiments have shown that the king penguin call is vital for individual recognition [41, 42]. The display call is highly stereotyped within individuals but highly variable between individuals. For mate or parent identification, king penguins perform a complex analysis of the call, using both frequency modulation and the beat pattern of the two voices [42]. Given that a breeding king penguin couple takes turns to take care of the egg or later the chick, there is a high selection pressure on individuals to find their mate and later locate the chick in the colony among thousands of conspecifics. During calling, king penguins adopt a particular posture: they raise their beaks slowly to a vertical position and stretch their necks to the fullest extent and emit a call (Fig. 6a). This posture limits signal-to-noise ratio reduction caused by the screening effect of the bodies of the birds gathered in dense flocks in the breeding areas [43].

The king penguin display call consists of a succession of frequency modulated syllables with two simultaneous series of harmonically related bands of slightly different frequencies and their respective harmonics, a “two-voice signal” [41, 44, 45]. It is likely that the underlying mechanism of the ability to produce two related frequency bands in king penguins lies primarily in the anatomical structure of the syrinx [44, 45] and can neither be actively modulated nor attributed to nonlinear phenomena, such as subharmonics or biphonation [46].

Detailed knowledge about the vocal tract anatomy of penguins however is generally lacking. The first study conducted by Meckel [47] on the anatomy of the vocal tracts of three specimens of African penguins *Spheniscus demersus*, reports a septum of the trachea that became caudally thicker and was strongest just before the bifurcation of the two bronchi. Watson [48] described the tracheal septum as being present in *Procellariidea*, but not in the Southern rockhopper penguin *Eudyptes chrysocome* or the Little penguin *Eudyptula minor*. Zeek [49] investigated the vocal apparatus of African penguins and confirmed the presence of the septum calling it a “double trachea”. Despite the presence in some penguin species, king and emperor penguins *A. forsteri* have been reported to “appear” to have no tracheal septum [50]. However, Davenport and colleagues [50] also suggested that penguins’ tracheae need to be studied more thoroughly to draw conclusions on the functional adaptation of a tracheal septum.

Here, we present the detailed anatomy of the entire vocal tract of king penguins, who have previously been reported to produce two-voiced sounds [41, 42]. We describe syringeal skeleton, soft tissues and cartilaginous parts using high-resolution micro-computed tomography (μCT) and analyse structural elements in three-dimensional (3D) reconstructions.

## Results

### Vocal tract anatomy

We find no major differences in the gross anatomy of vocal tracts between male and female king penguins. Therefore, the anatomical features described subsequently are only shown exemplarily in the male.

**Fig. 1:**
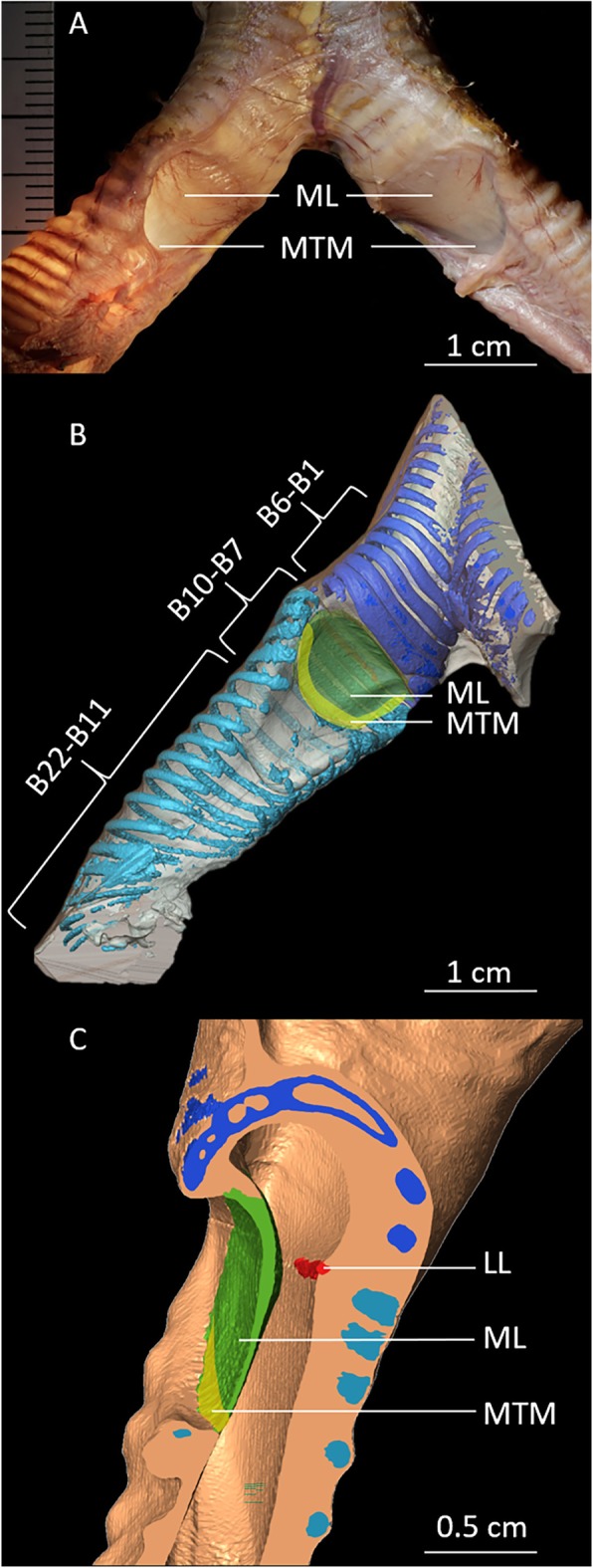
Syrinx anatomy of the king penguin. **a**: Image of a male king penguin’ syrinx at the bifurcation of the trachea in the two primary bronchi (dorsal view). Internal illumination of the left bronchus reveals the pronounced vasculature of the medial labium (ML) and the medial tympaniform membrane (MTM). **b**: μCT 3D visualization of the right bronchus showing osseous tracheal rings and osseous (dark blue) and cartilaginous (light blue) bronchial rings surrounding the vibratory membranes. B1-B5 are completely ossified bronchial full rings, while B6 is a C-shaped half rings. B7-B22 are C-shaped cartilaginous half rings. B5-B11-B22: C-shaped cartilaginous rings of the main bronchus. **c**: μCT 3D visualization of a parasagittal section through the left side of the syrinx showing the lateral labium (LL), the ML, the MTM, bronchial cartilaginous rings (turquoise) and ossified bronchial rings superior to the vibratory tissue (dark blue)

The king penguin has a bronchial syrinx (Fig. 1a), with a medial labium (ML), lateral labium and a medial tympaniform membrane (MTM) as vibratory tissues (Fig. 1). The vascularization in the ML is considerably more prominent than in the MTM that contains almost no macroscopically visible blood vessels (Fig. 1a, left side). Each primary bronchus has a total of 22 rings of different shapes and degrees of mineralization (Fig. 1b). Beginning at the bifurcation the bronchial rings B1-B6 are ossified. B1-B4 superior to the labia are full rings. B3 and B4 are partially fused, dorsomedially flattened and here of a comparatively large circumference. Two half-rings follow, B5 and the rudimentarily developed B6, which are partially fused at their free ventromedial endings. The remaining bronchial rings, B7-B22, are cartilaginous. The first four, B7-B10, are half-rings and attached to the vibratory tissues, the ML and the MTM. Twelve C-shaped cartilaginous rings follow up to the hilum of the lung.

**Fig. 2:**
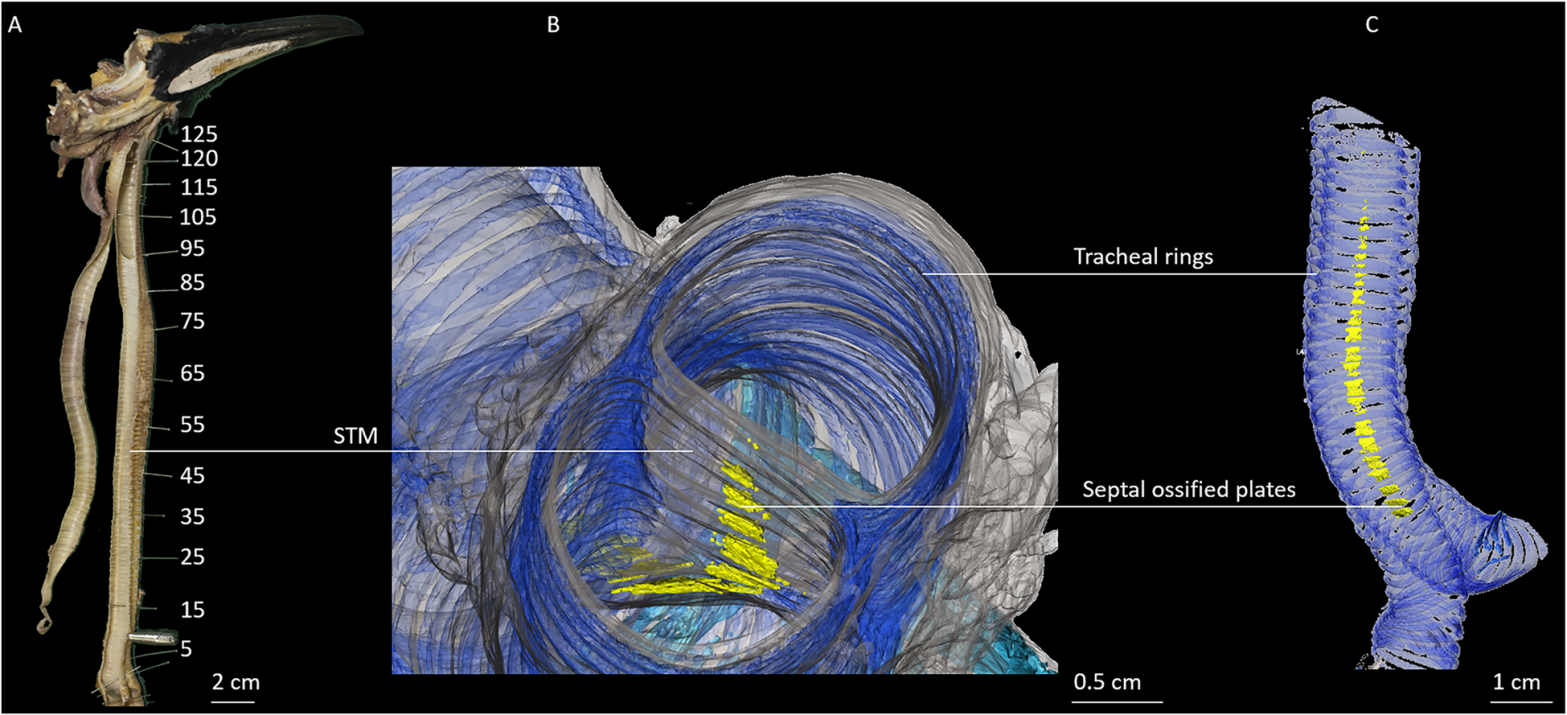
King penguin trachea and septum trachealis medialis (STM) separating the trachea into two lumina. **a**: Image of the upper vocal tract of a male king penguin. The right side of the trachea is removed and put aside to expose the medial septum. The STM originates at the bifurcation of the trachea into the two primary bronchi and stretches to the 94th/96th tracheal ring, 6 cm below the larynx. Tracheal rings are numbered 1–125. **b**/**c**: 3D reconstructions of trachea, syrinx and bronchi showing different densities of the trachea and STM. Blue indicates ossified tracheal and bronchial rings. Yellow depicts ossified plates within the connective tissue (grey) of the tracheal septum. **b**: Craniocaudal view into the double lumen trachea showing the STM separating the two tracheal tubes and the irregular ossified plates (yellow) segmentally lined up within the septum. **c**: Lateral view of the trachea and the ossified plates of the STM

The trachea is separated by a septum: septum trachealis medialis, (STM; Fig. 2a, b). The STM extends over 21 cm of the 27 cm long trachea (≈ 78%). The STM begins at the bifurcation of the trachea and ends 6 cm below the larynx between the 94th/96th rings (Fig. 2a). The septum consists mainly of soft elastic tissue, but in its caudal third contains ossified plates located at the height of the tracheal rings 6 to 21 (Fig. 2b, c).

**Fig. 3:**
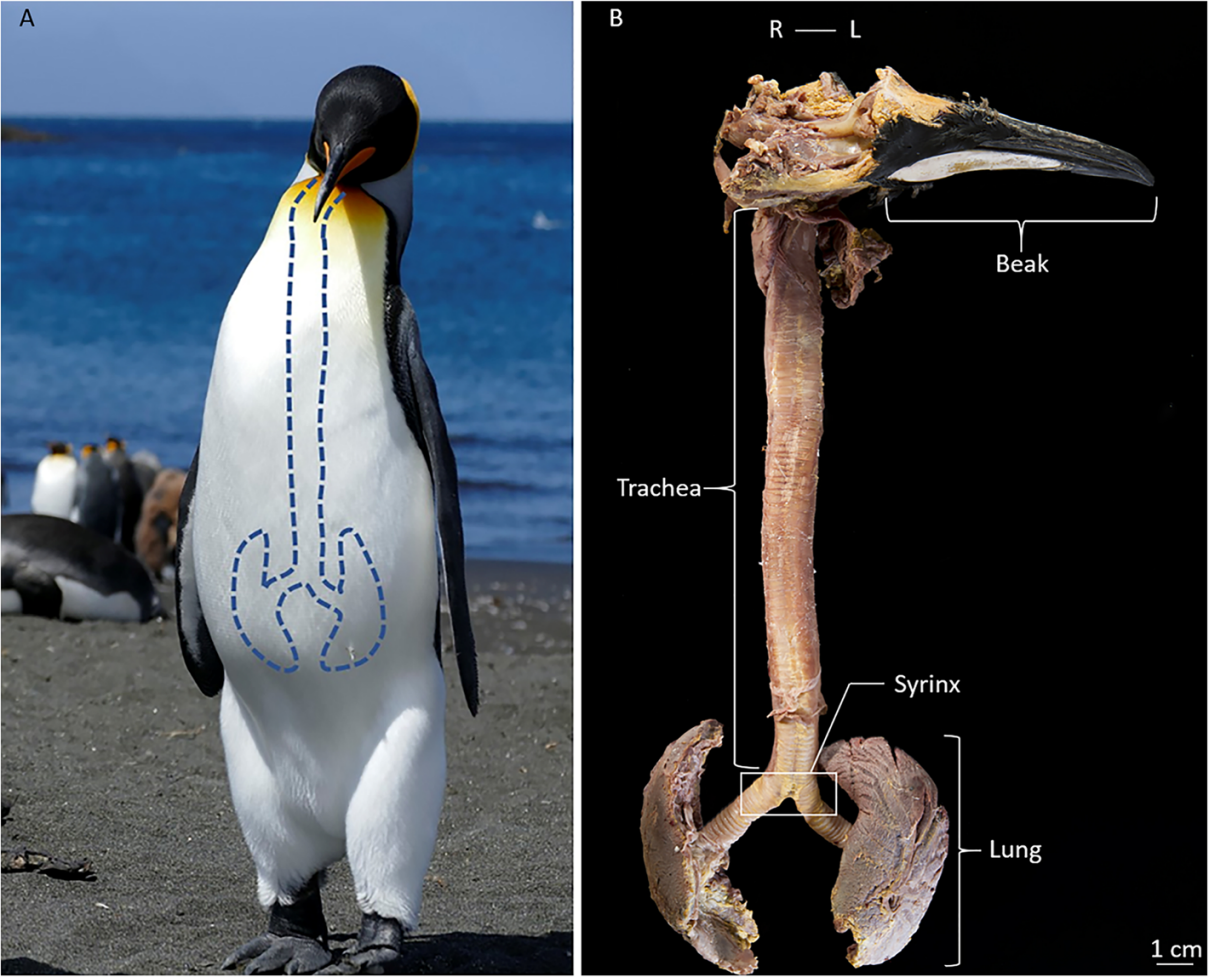
General anatomical structure of the king penguin vocal tract. **a**: Photograph of a male adult king penguin with a schematic drawing of the vocal tract to illustrate its location in the body. **b**: Image of the entire vocal tract of a male king penguin including beak, upper respiratory tract, trachea, syrinx, lower respiratory tract with main bronchi and lungs. White box indicates the position of the bronchial type syrinx. (R = right, L = left)

The lungs are indented dorsally by the vertebral ribs. The male trachea shows 125 partially ossified cartilaginous rings (Fig. 3a). It is elastic and has a length of 27 cm in the relaxed position in both males. The trachea of both male and female king penguins is not looped but lies straight in the thorax (Fig. 3b). While the overall structure of the vocal tract appears to be the same for males and females in our small sample group, we find slight differences in tracheal length between the sexes (Table 1).

**Table 1:** Size measurements of two male and two female vocal tracts

Sex	Trachea length (mm)	Diameter Trachea caudal end (mm)	Diameter mid Trachea (mm)	Diameter Trachea cranial end (mm)	Right ML length (mm)	Left ML length (mm)	∆ ML (mm)
Male 1	270	17.6	21.9	23.0	19.3	19.1	0.2
Male 2	270	18.9	21.6	21.0	20.6	20.5	0.1
Male 3	NA	NA	NA	NA	18.1	18.5	- 0.4
Male 4	NA	NA	NA	NA	18.0	17.1	0.9
Female 1	295	18.6	20.0	21.1	19.7	19.5	0.2
Female 2	275	18.2	19.6	20.5	18.4	19.0	- 0.6

ML = medial labium. The total trachea length is reported as well as trachea diameters measured at three points. Delta ML is the difference between right ML and left ML. The trachea of males 2 and 3 were cut prior to the size measurements which are therefore not available for those two samples

**Fig. 4:**
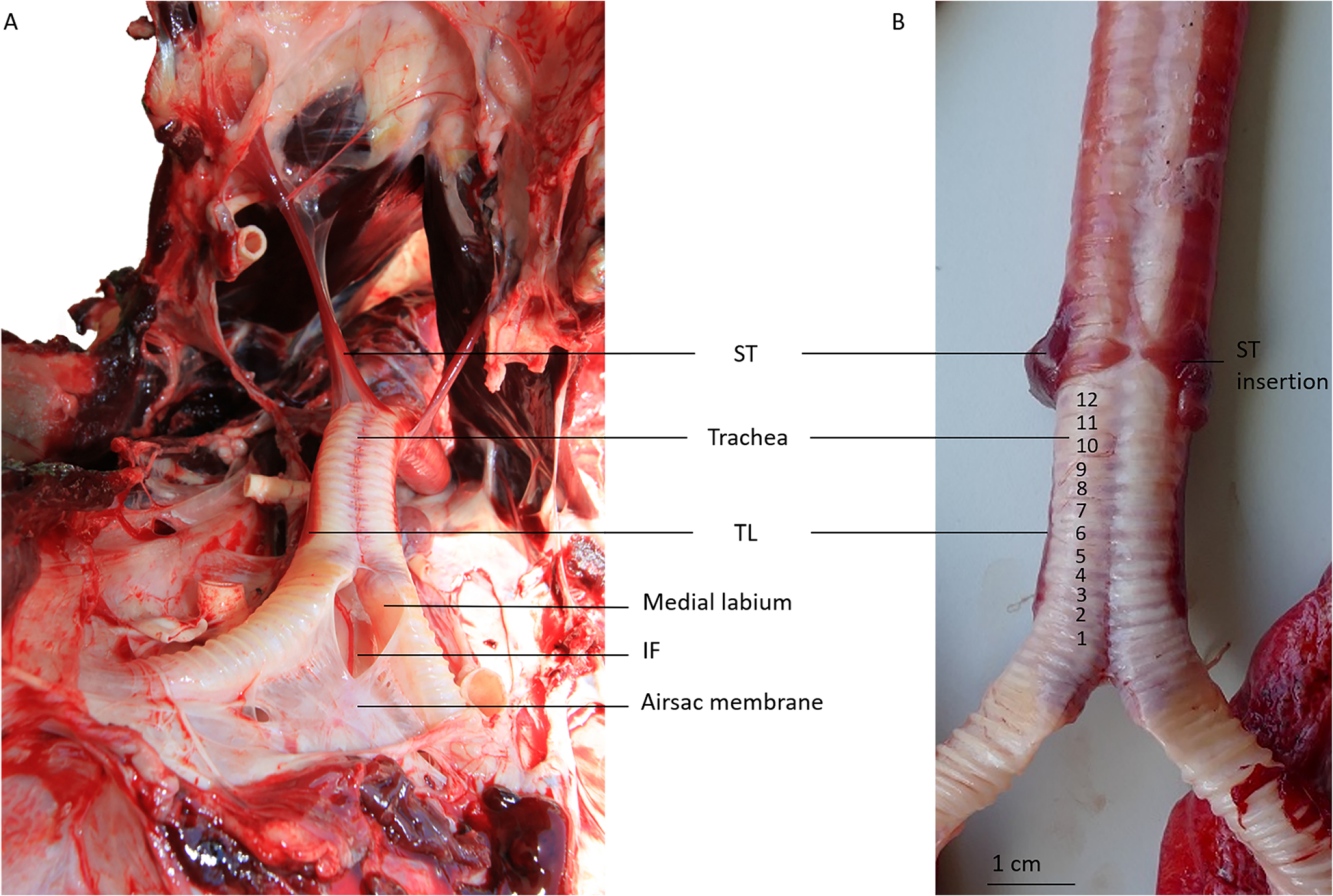
Syrinx location in situ and connected structures of a male king penguin. **a**: Craniodorsal view in situ: the sternum is lifted upwards. The right tympaniform membrane is covered by the right lateral side of the right bronchus. Note that the heart has been removed to allow better visibility of the bronchi. **b**: Ventral view of the syrinx ex vivo. ST = Sternotracheal muscles, TL = Tracheolateral muscles, IF = Interbronchial Foramen. Numbers indicate tracheal rings

The tracheolateralis (TL) muscle runs alongside the trachea (Fig. 4a,b) and attaches between the 2nd and 3rd tracheal rings, well before the skeletal elements directly connected to the vocal membranes (Fig. 4a, b). The sternotrachealis (ST) muscle inserts ventrally at the lower trachea between the tracheal rings 11 and 14 (Fig. 4b).

**Fig. 5:**
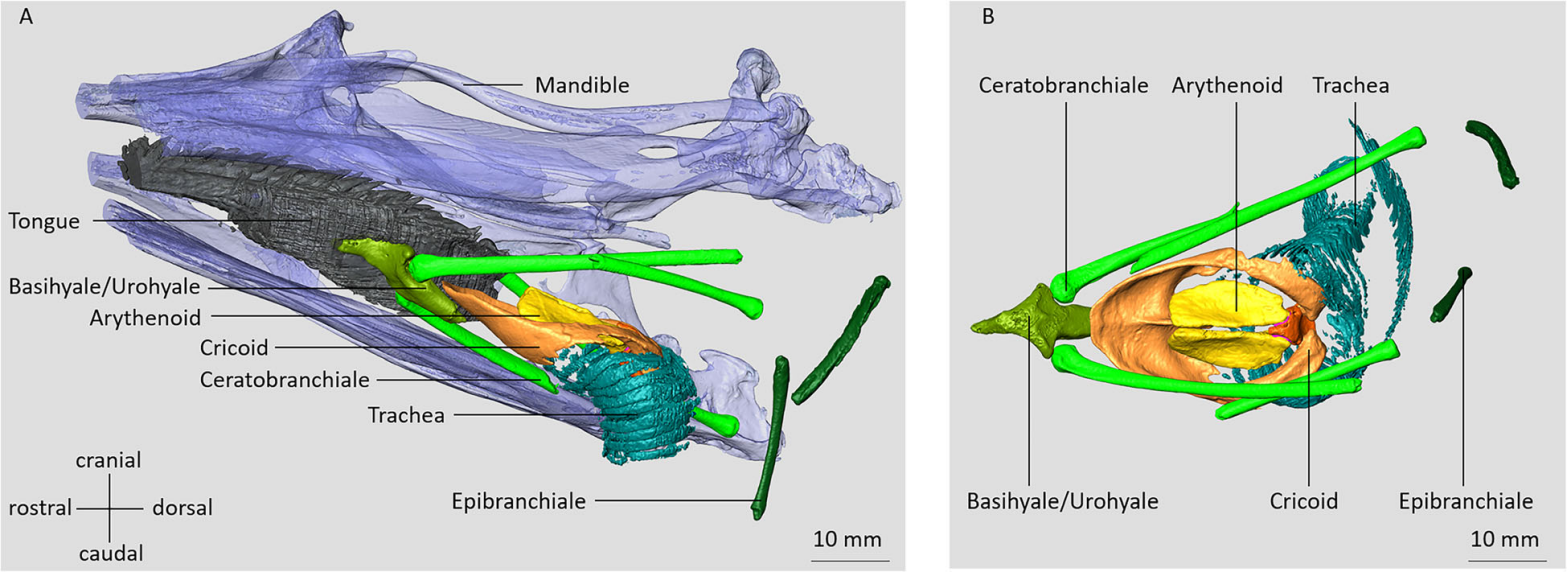
Surface rendering of μCT data of a male king penguin skull and upper vocal tract. **a**: Skull, tongue, hyoid, larynx and upper trachea seen from laterocaudal, left side. **b**: Craniocaudal view of the hyoid apparatus and the larynx. Note that both ceratobranchial bones are fractured and both epibranchiale, connected through joints with the ceratobranchiale in the live bird, were dislocated in this specimen. At the left edge of the figure the tip of the beak is missing due to limitations of the physical space in the CT-scanner

The 3D reconstruction and analysis of the upper vocal tract, specifically of the head and throat (Fig. 5), show no particular differences or anomalies as compared to other birds [51, 52]. The basihyale is located at the base of the tongue and extends into the urohyale. Basihyale and urohyale are fused, with an articulated connection to the paired ceratobranchiale. The articulated connection is located at the transition of basi- and urohyale. The basihyal, urohyal, paired ceratobranchial and epibranchial bones are all part of the hyoid apparatus [51]. The basihyal bone and urohyal bone form the paraglossale, i.e. the structure which is connected to the base of the tongue. The tongue is a rigid structure that dorsally contains lingual filiform-like papillae [53]. The epibranchial bones are arched, bilaterally paired bones that form the tip of the hyoid structure. They are connected by joints to the ceratobranchial bones, which were fractured in this specimen. Thus, their location does not correspond to their natural position. The ossified cricoid, arythenoids and procricoid form the larynx.

The two male specimens have a shorter overall trachea length while the females have a smaller overall trachea width (Table 1). For the four specimens, the trachea width increases from caudal to cranial on average by 16%. Length of left and right ML differs consistently between all six individuals with no obvious side dominance.

### Call analyses

Male and female king penguins differ significantly in the difference in frequency between the two voices (Welch’s *t*-test, *P* = 0.011; Table 2). We find the extent between the fundamental frequencies of the first and the second voices, i.e. F0_1_ and F0_2_, to be consistently higher for female king penguins with 49.0 ± 12.9 Hz than for males with 40.3 ± 11.5 Hz. There are no sex differences between F0_1_ and F0_2_ (Welch’s *t*-test, *P* > 0.05; Table 2). For the individual recognition process in king penguins, both the frequency modulation of the call and the beats generated by the interaction of F0_1_ and F0_2_ are equally important [41]. A smaller frequency extent generates a longer beat period. Males had a significantly longer period of beats than females (Table 2).

**Table 2:** Measurements and statistical comparison of the two voices of male and female king penguin calls

Variable	Males (*n* = 42)	Females (*n* = 19)	*P*-value	DF	t-value
F0_1_ [Hz]	423.2 ± 54.6	439.2 ± 41.7	0.217 n. s.	44.91	1.25
F0_2_ [Hz]	463.5 ± 55.6	488.2 ± 45.9	0.07 n. s.	41.74	1.82
Frequency extent F0_2_ [Hz] - F0_1_ [Hz]	40.3 ± 11.5	49.0 ± 12.9	0.011	59.00	2.62
Period of Beats (1/ frequency extent) [s]	0.027 ± 0.007	0.022 ± 0.006	0.009	38.01	−2.76

Mean ± standard deviation and results of Welch’s t-tests comparing the first (F0_1_) and the second voice (F0_2_) between males and females. The extent did not follow a normal distribution and we thus performed a generalized linear model with a gamma transformation

## Discussion

**Fig. 6:**
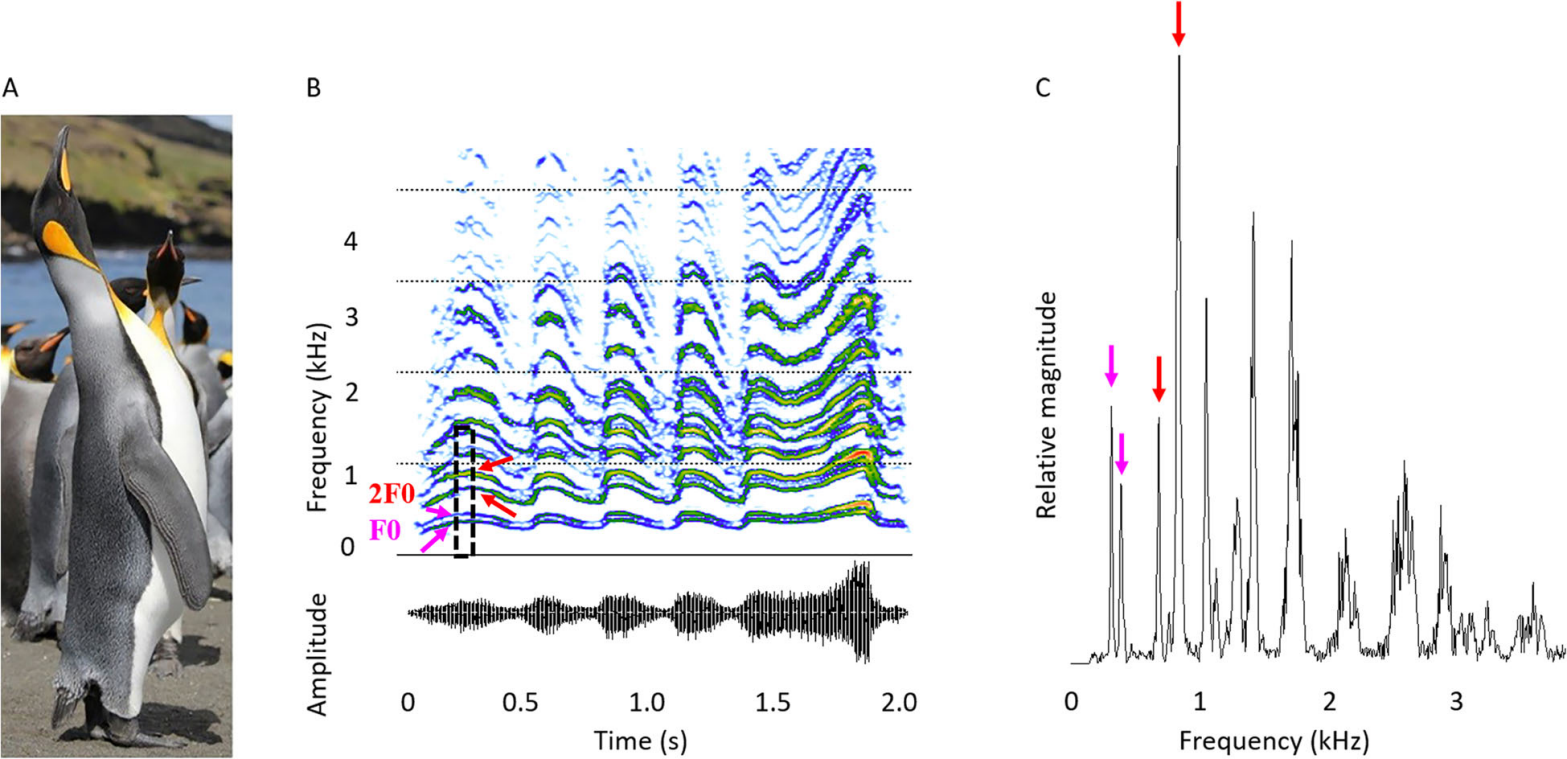
Body posture of vocalizing king penguin and spectrogram and power spectrum of a male call. **a**: Adult king penguin in the typical calling position emitting a call. **b**: Spectrogram (FFT length 1024, 98.43% overlap, Blackman window) of an adult male king penguin call. Below the oscillogram of the call. **c**: Amplitude spectrum of the call section marked with a dotted rectangle in **b** (duration: 0.1 s). **b** and **c**: F0 = Fundamental frequency (first harmonic), 2F0 = Second harmonic. The pink and the red arrows indicate the two voices for F0 and 2F0, respectively

We hypothesize that the ritualized calling position of king penguins (Fig. 6a), during which they do not move, results in the stability of the vocal output, i.e. the stereotypy of the frequency of the two voices. A similar conclusion was drawn by Favaro et al. [36] for African penguins. Using endoscopic imaging in living birds, Goller and Larsen [22] observed that the phonation process in two songbird species was preceded by the contraction of the TL muscles leading to an upward movement of the syrinx and consequentially, the bronchial half-rings were pulled apart and the vibratory tissues stretched. It is possible that this mechanism applies for king penguins as well. Additionally, contraction of the TL would pull the ossified plates of the STM closer together causing the STM to stiffen. Furthermore, the calling position, i.e. stretching and pointing the beak upwards (Fig. 6a) enhances the propagation of the call in the crowded and noisy colony [54, 55]. The increased tracheal diameter from caudal to cranial likely increases the overall amplitude of the produced sound and therefore improves the chances to be heard by the target conspecific. We therefore hypothesize that this calling position has evolved to enhance sound pressure level and signal propagation and does not serve size exaggeration [56].

The key feature that allows both male and female king penguins to produce two simultaneous fundamental frequencies is their bronchial syrinx type. Using a mechanical model of the avian syrinx, Elemans et al. [57] showed that the fundamental frequency of the vocal output highly correlates with the fundamental oscillation frequency of the vibrating membrane. In general, the membranes’ fundamental frequency is determined by the elastic properties, size and tension of the membranes [20, 57]. Düring et al. [18] showed that increasing the length of the medial labia in various directions through muscle activation results in higher fundamental frequencies. Given the absence of intrinsic syringeal musculature, the extent between the two voices (F0_1_-F0_2_) in the king penguins’ call could be determined through the different sizes of the vibratory tissues on each side of the syrinx. Lateralization of the syrinx as the source of acoustic features has been proposed in numerous studies [29, 30, 58, 59]. The exact role of the extrinsic ST and TL muscle remains unclear, however, it seems possible that the activation of the TL leads to passive stretching of the vibratory tissues and thus also influences the two-voice frequencies [60]. Future ex vivo studies [9] or biomechanical models based on tissue properties [18, 61] would be necessary, to reveal the precise mechanism and control possibilities of the two voices of king penguins. Another important aspect to consider is the vibration frequencies as a result of source-filter interaction, where non-vibratory structures in the vocal tract interact with the vibratory tissues leading to spectrally complex sounds [62]. Sound pressure waves are generated by the vibratory tissues and are reflected, i.e. coupled in the vocal tract and travel back to the oscillating tissues [63]. The upper vocal tract acts as a filter for the sounds produced by the vibratory tissues (source) and the acoustic coupling of source and filter influences the acoustic properties of vocalisations (e.g. [37, 64, 65]).

The size, location and type of tissue of the STM as part of that filter suggests influences on the vocal output as well. The STM could play an important role in the coupling of each sound source with either side of the vocal tract, and the resulting delayed acoustic feedback could influence the spectral characteristics of the king penguin call. Furthermore, as this coupling process happens for the two voices F01 and F02 on each side of the king penguin syrinx separately, the STM could function as a “barrier”, preventing F01 and F02 from mixing for most of the length of the trachea.

Tracheal septa have been reported to be present in little penguins [66], African penguins [37], dugongs *Dugong dugon* [49], and leatherback turtles [50]). Reports on leatherback turtle and dugong vocalisations are scarce and provide little or no evidence for individual vocal signatures (leatherback turtles [67, 68], dugongs [69]). Convincing evidence for two voices is thus only available for king penguins [41], emperor penguins [44] and African penguins [70]. The STM could, for example, play an important role in keeping the two independently produced sounds separated until the cranial end of the trachea. Therefore, it might allow or enhance the psychoacoustic recognition of the two synchronously produced voices. Favaro et al. [36] used computational models of African penguin vocal tracts and showed that the difference between air resonance in a single versus a two-tube model was related to a variation in formant position and dispersion. However, the authors simplified their model assuming that “the trachea is divided by a septum for all its length”, which is not the case, neither in African [49] nor in king penguins. Still, formant frequencies have been shown to be individually distinct and might encode individual identity, e.g. in African penguins [37] and in corncrakes *Crex crex* [71].

Given that a tracheal septum has also been reported for other aquatic species [36, 49, 50], one needs to consider the possibility that it might have originally evolved for a different, non-acoustic function, for example baroprotection. Ponganis et al. [72] determined individual body densities and lung/air sac and body volume of Adélie *Pygoscellis Adeliae*, king and emperor penguins and concluded that diving penguins probably reduce the air volume of the parabronchial, tracheobronchial and lung air spaces to prevent pulmonary barotrauma. The STM could have a similar function as to prevent the collapse of the trachea. Another hypothesis is that the septum allows for simultaneous feeding and breathing in diving species as suggested by Davenport et al. [50], who reported a tracheal septum in leatherback turtles *Dermochelys coriacea*.. The tracheal septum might play a vital role in preventing the compression of the lower part of the trachea caused by the food-filled oesophagus and hence might facilitate breathing.

## Conclusion

Male and female king penguins do not differ substantially in basic anatomical features of the vocal tract. Both sexes possess a bronchial type syrinx allowing for the simultaneous production of the two voices featured in the king penguins characteristic display calls. The difference in size between left and right labia results in an offset between the two fundamental frequencies, i.e. the beat that encodes for individuality in *Aptenodytes*. The STM separating the trachea into two lumina may further favour the production or psychoacoustic recognition of the two voices, while additionally aid in stabilizing the trachea during prey ingestion. The increased diameter from caudal to cranial in connection with the calling position adopted by king penguins during vocalising is likely an adaptation to improve the propagation of this important signal of individuality in the environment of a noisy seabird colony.

## Methods

### Sample collection

To study the detailed anatomy of the king penguin vocal tract, six specimens were collected in the king penguin colony of “La Baie du Marin” at Possession Island, Crozet Archipelago (46°25’S, 51°45’E), between November 2015 and April 2016. Vocal tracts were dissected from four males and two females that were found freshly dead at the periphery of the colony. While the exact age of the birds is unknown, plumage coloration indicates that all individuals were adults, i.e. at least four years or older. Sex was determined for all birds through macroscopic identification of the gonads during dissection, following Hocken [66]. Vocal tracts were removed in toto, i.e. head (without the orbital region, brain case and brain), beak, tongue, hyoid bones, trachea, syrinx, lungs and attached muscles as one connected structure (Fig. 3b). Vocal tracts were stored in 4% phosphate-buffered Paraformaldehyde (PFA) solution at + 4 °C.

### Ex situ measurements

Tracheal diameter was measured at three different levels, (i) cranial just below the larynx, (ii) the middle of the trachea and (iii) caudal just above the bronchial bifurcation. Tracheal length was measured between the bronchial bifurcation and the last tracheal ring just below the larynx. Width measurements of medial labia were taken at the inside of bronchial bone B7. All measurements were performed ex situ on fixed vocal tracts.

### Soft tissue counterstaining and sample preparation for Micro-computed tomography

To visualize soft tissues [73, 74], the syrinx of a female and the entire vocal tract of a male and a female specimen were counterstained with 10% Lugol’s solution (Sigma Aldrich, St. Louis, MO, USA) for up to 7 days. Lugol’s solution was replaced every two days until the iodine coloration of the staining liquid remained stable. To prevent samples from moving during image acquisition we prepared customized polystyrene forms for each sample. Forms were prepared to fixate each sample in a position resembling in vivo positioning as close as possible. Each form and the containing sample were subsequently placed in separate Polymethyl methacrylate (PMMA) tubes for μCT scanning. Prior to scanning, we added a few ml of water to the tubes to prevent interference due to changes in humidity caused by x-ray related temperature fluctuations.

Scans were performed using a Phoenix nanotom m cone beam μCT scanner (GE Measurement and Control, Wunstorf, Germany). Scans for the entire vocal tracts were conducted using three slightly overlapping field of views (FOV), each acquiring 1600 projections during a 360° rotation at a voltage of 120 kV and 100 mA current. The female syrinx dataset was acquired as one individual FOV scan with the same settings. All scans were performed using a tungsten (“standard”) target with an additional 0.1 mm aluminium filter. Voxel size of the female syrinx was 0.04 μm and of the male syrinx 0.05 μm.

### Visualization and analysis of μCT data

Micro-CT raw data were reconstructed and filtered using the software phoenix datos|× 2 (GE Sensoring & Inspection Technologies GmbH, Germany). Subsequently, they were cropped and converted to 8bit using VGStudio MAX 2.2 (Volume Graphics, Heidelberg, Germany). Stitching of multiple data sets, threshold based plus additional manual segmentation, surface generation of the μCT data were performed with Amira 6.0.1 (FEI Visualization Sciences Group, Burlington MA, USA), following the protocol of Ruthensteiner [75].

### Call recordings and analyses

King penguin vocalisations were recorded also in the king penguin colony of “La Baie du Marin” (approx. 16,000 breeding pairs [76]) between November 2015 and April 2016. Display calls of 42 males and of 19 females were recorded using an omnidirectional Sennheiser K6-me62 microphone (frequency response: 20–20,000 Hz ± 2.5 dB) mounted on the end of a 2 m rod held by a human observer and connected to a Marantz PMD 661 digital recorder (frequency response: 20 Hz-24 kHz ± 1 dB, sampling frequency: 44.1 kHz), the microphone being at approximately 1 m distance from one side of the bird’s beak when it assumed the typical calling posture.

We measured the overall frequency of the two fundamentals and of their difference in frequency in syllables of males and females. As display calls of king penguins are highly redundant, consisting of successive syllables with a repetition of the same individual information many times (one syllable is sufficient to elicit recognition [77]), our frequency measurements were taken on the first syllable only, in a 0.1 s time window of the syllable (see Fig. 6b, c).

Signals were analysed using Avisoft SASLab Pro (version 5.2.09, [78]) and down-sampled prior to analysis to 22.05 kHz and high-pass filtered at 0.20 kHz to remove the background noise. Frequency measurements of the two fundamentals F0_1_ and F0_2_ were done on the spectrum (Fast Fourier Transform (FFT) length of 1024, Blackman window). In cases where we had recorded and measured the two voices of more than one call (N_females_ = 12, N_males_ = 28), the measurements were averaged for each individual. We analysed the two-voice frequencies for a total of *N* = 42 males and *N* = 19 females. To compare two-voice measurements between the sexes, we performed (1) Welch’s *t*-tests on the first (F0_1_) and second (F0_2_) voices and on the beats, i.e. 1/(F0_2_- F0_1_), and (2) a generalized linear model (family = gamma) on the non-normally distributed frequency extent, i.e. F0_2_ - F0_1_.

## Data Availability

Data availabilityThe datasets used and/or analyzed during the current study are available from the corresponding authors on reasonable request.
